# Fluoroquinolone Use and Risk of Pneumothorax in Adults Hospitalized With Community Acquired Pneumonia: A Retrospective Cohort Study

**DOI:** 10.1111/crj.70212

**Published:** 2026-07-01

**Authors:** B. S. Steven Yi, B. S. Saurav Sumughan, M. D. Jessica Cobb, Erika J. Yoo

**Affiliations:** ^1^ Sidney Kimmel Medical College Philadelphia Pennsylvania USA; ^2^ Department of Medicine Thomas Jefferson University Philadelphia Pennsylvania USA; ^3^ Division of Pulmonary, Allergy and Critical Care Medicine Thomas Jefferson University Philadelphia Pennsylvania USA

**Keywords:** fluoroquinolones, pneumonia, pneumothorax

## Abstract

**Objectives:**

The objective of this study is to examine the relationship between fluoroquinolone exposure and pneumothorax risk in patients hospitalized with pneumonia.

**Methods:**

This retrospective cohort study used the TriNetX US Collaborative Network. We defined two mutually exclusive cohorts of hospitalized adults based on exposure to antibiotic classes commonly used to treat community acquired pneumonia: fluoroquinolone (ciprofloxacin, moxifloxacin, levofloxacin, and ofloxacin) versus nonfluoroquinolone (amoxicillin, azithromycin, ceftriaxone, and doxycycline). Patients with connective tissue disorders were excluded. The primary outcome was the occurrence of pneumothorax within 60 days. We used Cox proportional hazards regression to estimate hazard ratios (HRs) adjusted for demographics, comorbidities, and markers of illness severity.

**Results:**

Among 1 140 106 eligible patients, 86 814 (7.6%) received fluoroquinolones. Fluoroquinolones as a class were not associated with risk of pneumothorax (HR 1.29, 0.95–1.76, *Q =* 0.21). Among individual fluoroquinolones, Ciprofloxacin alone was associated with an increased risk of pneumothorax (HR 2.26, 95% CI 1.38–3.69, *Q <* 0.01). Pooled nonfluoroquinolone antibiotics were associated with a modestly reduced hazard of pneumothorax (HR 0.79, 95% CI 0.69–0.91, *Q* < 0.01), although this effect was not consistent across individual agents.

**Conclusions:**

Exposure to fluoroquinolones does not appear to impact the risk of pneumothorax in hospitalized adults with pneumonia. Only ciprofloxacin was associated with increased risk of pneumothorax. Future research in both clinical and experimental settings may help elucidate whether the mechanism by which fluoroquinolones affect the pulmonary pleura can vary by acute disease state. Our findings suggest that antibiotic selection for pneumonia should be guided by clinical appropriateness rather than attributable risk for pneumothorax.

## Graphical abstract

We conducted a retrospective multicenter cohort study to evaluate the association between fluoroquinolone administration for inpatients with pneumonia and 60‐day risk of pneumothorax. We found no association between fluoroquinolones and pneumothorax compared to other common antibiotics for community acquired pneumonia. Heterogeneity was observed among individual agents. These findings support antibiotic selection based on clinical appropriateness rather than concern for pneumothorax.

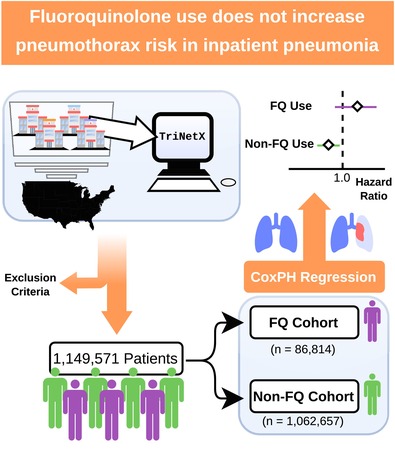

## 1 Introduction

Pneumonia and its complications remain among the leading causes of hospitalization and death in the United States. Pneumonia is estimated to account for 4% of all adult hospitalizations and an in‐hospital case fatality of roughly 7%–8% among pneumonia‐associated admissions [1]. Although many patients can recover without incident, a subset can develop complications, including secondary pneumothorax, parapneumonic effusion, or empyema.

The frequency of pneumothorax in pneumonia is low, typically less than 2% for most types of pneumonia, with a rate of 2.2% in bacterial pneumonia based on a large autopsy population. However, it can rise as high as 38% in those with aspiration pneumonia requiring ventilator support [2, 3, 4, 5]. Pneumothorax in the setting of pneumonia is associated with significantly worse outcomes, including increased mortality, higher rates of intensive care unit (ICU) admissions and mechanical ventilation, and more extended hospital stays compared to those without pneumothorax [3, 6, 7, 8].

The pathogenesis of pneumothorax is thought to involve a combination of factors, including existing pleural abnormalities, environmental exposures, airway inflammation, and abnormal matrix remodeling activity [9]. The pleura contains loose connective tissue composed primarily of extracellular matrix (ECM), whose structural integrity depends largely on matrix metalloproteinases‐regulated collagen turnover [10]. Matrix metalloproteinases (MMPs) are key regulators of ECM remodeling, tissue turnover, and local inflammatory response. MMPs play a pivotal role in the pathogenesis of pneumothoraces, as the degradation of ECM increases pleural leakage of air into the pleural space (pleural porosity) and facilitates the formation of blebs and bullae [11, 12]. In the case of pneumothorax specifically secondary to pneumonia, airway inflammation is a significant contributor [13]. However, the impact of antibiotics, if any, on the occurrence of pneumothorax remains unclear.

Ex vivo evidence demonstrates that certain antibiotics can modulate the remodeling of the ECM. For instance, fluoroquinolones appear to promote MMP activity by myofibroblasts [14]. Doxycycline appears to decrease MMP activity by fibroblasts in certain experimental conditions [15]. Clinical data reflect the effects of certain antibiotics on the pathogenesis of disorders affecting collagen‐rich tissues throughout the body; fluoroquinolones, in particular, are associated with tendonitis and tendon rupture, aortic aneurysm and dissection, retinal detachment, and bowel perforation [16, 17]. However, other commonly used antibiotics in the management of community‐acquired pneumonia, including amoxicillin, azithromycin, doxycycline, and ceftriaxone, have not demonstrated such an association [16, 18, 19].

Furthermore, MMPs play a key role in the pathogenesis of pneumonia, as they are predominantly released by activated neutrophils, facilitating leukocyte migration, promoting ECM breakdown, and disrupting the alveolar‐capillary barrier [20]. MMP levels and activity reflect disease severity and pathogen virulence. This altogether highlights the proposed intricate interplay between elevated MMPs in the setting of pneumonia and antibiotic‐induced alterations in MMP activity, which could weaken pleural tissue during active pneumonia, potentially influencing susceptibility to pneumothorax.

Despite the established effects of certain antibiotics on collagen‐rich tissues elsewhere in the body, their potential role in pulmonary structural complications such as pneumothorax, particularly in the setting of pneumonia, has not been systematically investigated. The present study aims to evaluate the relationship between exposure to respiratory fluoroquinolones to treat community acquired pneumonia and the incidence of pneumothorax. We hypothesized that the use of fluoroquinolones would increase the risk of pneumothorax in patients hospitalized for this condition.

## 2 Methods

### 2.1 Data Source

We conducted a retrospective cohort study using deidentified data from the TriNetX US Collaborative Network [21], which aggregates electronic health records from academic medical centers, community hospitals, and outpatient clinics across the United States. The database contains patient‐level demographics, diagnoses, procedures, laboratory measurements, and medication orders collected during routine clinical care. Data are standardized to common ontologies and undergo periodic validation. All records are deidentified in compliance with the Health Insurance Portability and Accountability Act, and analyses are performed within the TriNetX secure platform. Because the study used only deidentified data, institutional review board approval and informed consent were not required. The study period spanned August 28, 2005, to August 28, 2025.

### 2.2 Patient Selection

**FIGURE 1:**
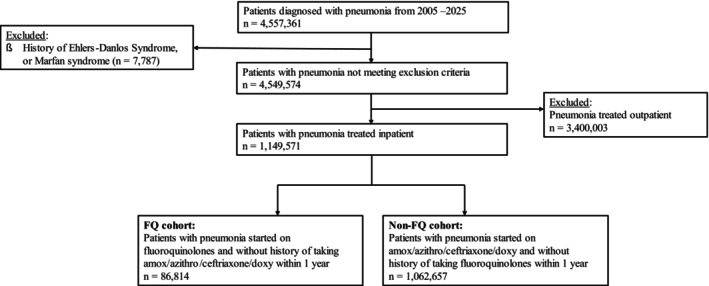
Cohort selection. Flow diagram showing the identification of adult patients diagnosed with pneumonia from 2005 to 2025, application of exclusion criteria, and final classification into fluoroquinolone (FQ) and nonfluoroquinolone (non‐FQ) treatment cohorts.

Patient selection is illustrated graphically in Figure 1. We identified adults (≥ 18 years) with an ICD‐10 diagnosis of pneumonia (J18*) who received one of the study antibiotics on the same calendar day as the pneumonia diagnosis and were hospitalized on the same date (index date). Two mutually exclusive exposure cohorts were defined using categories of antibiotics commonly used to treat community acquired pneumonia per guidelines put forth by the Infectious Diseases Society of America (IDSA) [22]. The fluoroquinolone (FQ) cohort included patients prescribed moxifloxacin (ATC J01MA14), levofloxacin (J01MA12), ciprofloxacin (J01MA02), or ofloxacin (J01MA01) without having used amoxicillin (J01CA04), azithromycin (J01FA10), ceftriaxone (J01DD04), or doxycycline (J01AA02) in the preceding year. The comparator (non‐FQ) cohort included patients prescribed amoxicillin, azithromycin, ceftriaxone, or doxycycline without the use of fluoroquinolones in the preceding year. Prescriptions could be issued in either inpatient or outpatient settings. To ensure exclusivity, patients with same‐day prescriptions from both categories were excluded. Patients with a history of Ehlers‐Danlos or Marfan syndrome were excluded.

### 2.3 Outcome Definition

The primary outcome was the first recorded pneumothorax (ICD‐10 J93) within 60 days after the index date. Traumatic pneumothoraces (ICD‐10 S.270‐S27.2) or traumatic subcutaneous emphysema (ICD‐10 T79.7) were excluded as outcomes. Follow‐up began on the index date and ended at whichever occurred first: pneumothorax diagnosis, 60 days elapsing, or the end of available data. Mortality as a secondary outcome was also reported for its potential to contribute to residual bias.

### 2.4 Covariates

Covariates were assessed on the same day of pneumonia diagnosis. The variables included were as follows:

- Demographics: age, sex, and race.
- Acute care indicators: critical care time (CPT 99291); arterial or venous blood gasses (ABG/VBG) taken (CPT 82803); emergency endotracheal intubation procedure (CPT 31500); and mechanical ventilation (5A0920Z).
- Pulmonary comorbidities: other specified chronic obstructive pulmonary disease (J44), emphysema (J43), asthma (J45), and interstitial lung disease (J84), history of pneumothorax (J93).
- Acute diagnoses: acute respiratory distress syndrome (J80), acute respiratory failure (J96.0), and sepsis (A41.9 and A41.89).
- Other comorbidities: nicotine dependence (F17), hypertensive diseases (I10–I15), chronic ischemic heart disease (I20–I25), heart failure (I50), and obesity (E66).

Race was obtained as reported by the electronic health record via TriNetX and included as a categorical variable to assess any racial bias in cohort selection. All covariates except age and race were entered as binary variables in the regression model. We created three age categories: < 65, 65–74, and ≥ 75 years. Race categories included White (reference), Black, and other.

### 2.5 Statistical Analysis

Baseline characteristics were summarized as counts and percentages for categorical variables and means with standard deviations for continuous variables. The mean difference in age between the cohorts was statistically tested with a two‐sample *t* test. Differences in any of the other covariates and incidence of pneumothorax were tested with two‐proportion *z* tests.

We performed multivariable Cox proportional hazards regression using the TriNetX analytics platform. This regression analysis was conducted to estimate hazard ratios (HRs) with 95% confidence intervals (CIs) for the time to pneumothorax within 60 days. The time period during which a patient is considered at risk begins on the index date and ends at the occurrence of pneumothorax, 60 days after the index date, or at the end of the recorded data. Independent variables included individual antibiotic exposure, demographics, comorbidities, and severity markers, as specified above. We conducted sensitivity analyses using 30‐ and 90‐day follow‐up windows. To further assess for residual confounding, we constructed a crude Cox and a 1:1 matched cohort using selected comorbidities and pneumothorax incidence.

Further postprocessing and data visualization were performed in Python [23] utilizing the libraries Pandas [24], SciPy [25], Matplotlib [26], and Forestplot [27]. Class‐wise HRs and 95% CIs were calculated using a fixed‐effects inverse‐variance weighted meta‐analytic approach [28]. The *p* values for these HRs were computed using two‐sided *z* tests with the null hypothesis that the HRs equal 1. The CI for the ratio of HRs between the two class‐specific HRs was derived using the Wald approximation on the log‐transformed scale [29]. The 95% CI was calculated using the following formula:

$${\mathrm{CI}}_{\left(1-\alpha \right)}=\exp\left[\left(\ln\left({\mathrm{HR}}_{\mathrm{FQ}}\right)-\ln\left({\mathrm{HR}}_{\text{non}-\mathrm{FQ}}\right)\right)\pm z_{\alpha /2}\sqrt{SE_{\mathrm{FQ}}^{2}+SE_{\text{non}-\mathrm{FQ}}^{2}}\right]$$

where *α* = 05, representing a 95% significance level; *CI*_(1‐*α*)_ is the $\left(1-\alpha \right)\times 100\%$ CI for the ratio of HRs; *exp* is the natural exponential function; *ln* is the natural logarithm; $z_{\alpha /2}$ is the *z* critical value from the standard normal distribution given the value of *α*; *HR*_*FQ*_ and *HR*_*non‐FQ*_ are the class‐wise HRs of FQ and non‐FQ use, respectively; and *SE*_*FQ*_ and *SE*_*non‐FQ*_ are the standard errors of the corresponding log‐transformed HRs.

This method assumes that the estimated log HRs follow an approximately normal distribution, with the variance of their difference equal to the sum of their squared standard errors. Applying the corresponding quantile from the standard normal distribution ($z_{1-\alpha /2}$) yields an approximate $\left(1-\alpha \right)\times 100\%$ CI for the ratio of HRs after exponentiation. A resulting 95% CI for the ratio of HRs that includes 1 indicates no significant difference.

To control the false discovery rate (FDR) in multiple comparisons, we applied the Benjamini–Hochberg procedure and recorded the FDR‐adjusted *p* values as *q* values. This procedure was performed one time each with two separate families: (i) baseline table *p* values and (ii) Cox covariate *p* values and composite class‐wise antibiotic *p* values. Statistical significance was defined as a two‐tailed *Q* ≤ 0.05.

## 3 Results

After exclusions, there were 86 814 patients in the FQ cohort and 1 062 657 in the non‐FQ cohort. Baseline characteristics are shown in Table 1. Patients receiving fluoroquinolones were, on average, older (75.3 ± 15.8 years) than those in the comparator group (64.7 ± 23.9 years) (*Q* < 0.01). The distributions of patient sex were similar. The FQ cohort had a higher proportion of White patients (69.0% vs. 62.8%) and a slightly lower proportion of Black or African American patients (15.0% vs. 15.5%). Comorbidities such as chronic obstructive pulmonary disease (COPD), hypertension, ischemic heart disease, and heart failure were more prevalent among fluoroquinolone recipients. Indicators of acute illness severity at index, including acute respiratory failure, acute respiratory distress syndrome, critical care services, and blood gas analysis, were more common in the non‐FQ group. Mortality was 14.9% in the FQ cohort and 14.1% in the non‐FQ cohort, respectively (*p* < 0.01).

**TABLE 1:** Patient characteristics.

	FQ cohort (*n* = 86 814)	Non‐FQ cohort (*n* = 1 062 657)	*p* value	*Q* value
Demographics				
Age	75.3 ± 15.8	64.7 ± 23.9	< 0.01	< 0.01
Male	42 582 (49.2%)	542 702 (51.1%)	< 0.01	< 0.01
Race				
White	59 730 (69.0%)	667 181 (62.8%)	< 0.01	< 0.01
Black	12 969 (15.0%)	165 079 (15.5%)	< 0.01	< 0.01
Other Race	14 115 (16.2%)	230 379 (21.6%)	< 0.01	< 0.01
Acute care indicators				
Critical care time	28 257 (32.6%)	420 433 (39.6%)	< 0.01	< 0.01
ABG/VBG	18 407 (21.3%)	265 960 (25.0%)	< 0.01	< 0.01
Intubation, endotracheal, emergency procedure	6862 (7.9%)	106 415 (10.0%)	< 0.01	< 0.01
Ventilation	7859 (9.1%)	108 196 (10.2%)	< 0.01	< 0.01
Pulmonary comorbidities				
History of pneumothorax	383 (0.4%)	2757 (0.3%)	< 0.01	< 0.01
Other COPD	29 233 (33.8%)	290 183 (27.3%)	< 0.01	< 0.01
Emphysema	8391 (9.7%)	108 969 (10.3%)	< 0.01	< 0.01
Asthma	13 017 (15.0%)	210 233 (19.8%)	< 0.01	< 0.01
Other interstitial pulmonary diseases	6096 (7.0%)	72 273 (6.8%)	0.27	0.29
Acute diagnoses				
ARDS	2793 (3.2%)	44 303 (4.2%)	0.02	0.02
Acute respiratory failure	32 624 (37.7%)	505 066 (47.5%)	< 0.01	< 0.01
Sepsis, unspecified organism	30 738 (35.5%)	370 259 (34.8%)	0.17	0.18
Other comorbidities				
Nicotine dependence	19 056 (22.0%)	251 715 (23.7%)	< 0.01	< 0.01
Hypertensive diseases	62 639 (72.3%)	685 635 (64.5%)	< 0.01	< 0.01
Chronic ischemic heart disease	31 229 (36.1%)	345 330 (32.5%)	< 0.01	< 0.01
Heart failure	34 272 (39.6%)	379 534 (35.7%)	< 0.01	< 0.01
Overweight or obesity	20 757 (24.0%)	283 614 (26.7%)	< 0.01	< 0.01

*Note:* Continuous variables (e.g., age) are reported as mean ± standard deviation. Categorical variables are presented as *number* (*percentage*) of patients within each cohort. Percentages are calculated using the total number of patients in the respective cohort as the denominator. Hypothesis testing is also shown for the difference between the two groups, and *p* values are reported. These *p* values were then adjusted for false discovery rate by the Benjamini–Hochberg procedure and displayed as *Q* values. Any values of *Q* ≤ 0.05 represent statistically significant values.

Abbreviations: ABG, arterial blood gas; ARDS, acute respiratory distress syndrome; COPD, chronic obstructive pulmonary disease; VBG, venous blood gas.

**FIGURE 2:**
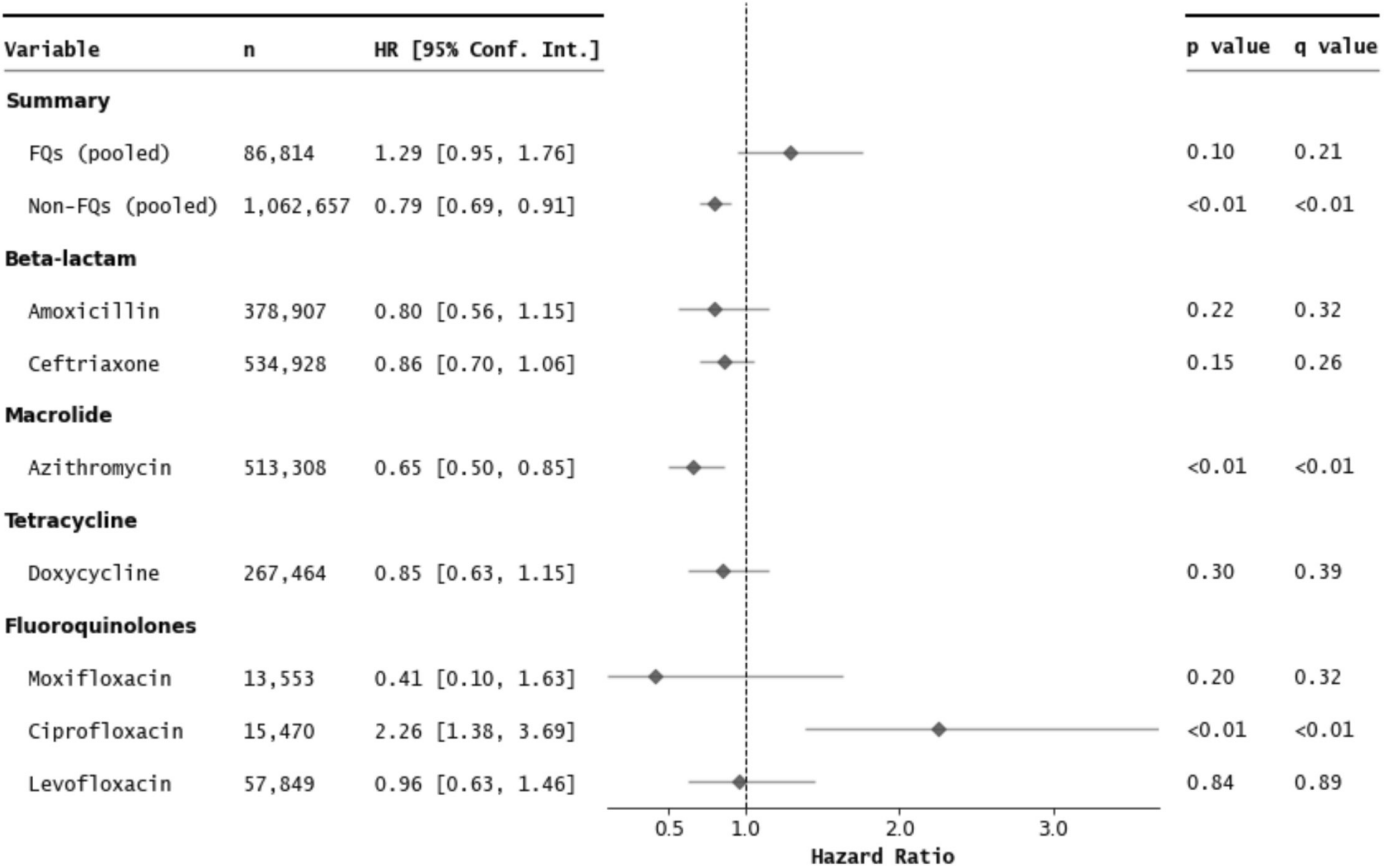
Forest plot of hazard ratios by antibiotic and cohort. Adjusted hazard ratios (HRs) and 95% confidence intervals (CIs) are shown for each cohort and individual antibiotic. The cohort portion of the forest plot is highlighted in gray. Points represent HR estimates, and horizontal lines represent 95% CIs. The *n* column lists the number of patients exposed to each antibiotic. The *p* values are unadjusted, and *Q* values are *p* values adjusted for FDR. Any values of *Q* ≤ 0.05 represent statistically significant values. The vertical dashed line at HR = 1.0 indicates no effect. HRs < 1 suggest a lower risk of pneumothorax compared with the reference group, whereas HRs > 1 indicate increased risk.

The primary outcome of interest, pneumothorax within 60 days, occurred in 302 (0.35%) patients in the FQ cohort and 5683 (0.54%) in the non‐FQ cohort. As a composite calculated after fitting the Cox regression for individual antibiotics, there was no association between FQ use and PTX risk within 60 days (HR 1.29, 0.95–1.76, *Q =* 0.21). Among individual quinolones, Ciprofloxacin was associated with an increased risk of pneumothorax (HR 2.26, 95% CI 1.38–3.69, *Q <* 0.01), but moxifloxacin (HR 0.41, 95% CI 0.10–1.46, *Q =* 0.32) and levofloxacin (HR 0.96, 95% CI 0.63–1.46, *Q =* 0.89) were not. Patients treated with ofloxacin had no pneumothorax events within the 60‐day window after the index date (Figure 2).

The 95% CI for non‐FQ use (HR 0.79, 95% CI 0.69–0.91, *Q <* 0.01) does not overlap with that of the FQ group (see Figure 2). The ratio of the class‐wise HRs of FQ use versus non‐FQ use was statistically significant at the 95% significance level (ratio of HRs: 1.63; 95% CI 1.16–2.29). Thus, the clinical risk profile associated with the use of fluoroquinolones was statistically significantly different from that of nonfluoroquinolone choices. The individual antibiotics constituting this group showed mixed results. For instance, azithromycin showed a significant decrease in hazard (HR = 0.65, 95% CI 0.50–0.85), whereas amoxicillin, ceftriaxone, and doxycycline did not show a significant association (Figure 2).

Markers of acute illness severity at the index encounter were strongly associated with the development of pneumothorax. Of the acute care indicators, critical care time and ABG/VBG sampling were associated with a higher risk of pneumothorax. Emphysema and interstitial pulmonary diseases were pulmonary comorbidities associated with increased risk of pneumothorax. Notably, asthma was associated with a reduced risk of pneumothorax. Remote history of PTX as a predictor of new PTX incidence resulted in failure of model convergence. More detailed HRs and significance levels are shown in Table 2.

**TABLE 2:** Hazard ratios for pneumothorax risk.

	Hazard ratio (95% CI)	*p* value	*Q* value
Demographics			
Age	0.998 (0.997–0.999)	< 0.01	< 0.01
< 65 years	3.094 (1.286, 7.441)	< 0.01	0.02
65–75 years	2.564 (1.065, 6.174)	0.03	< 0.05
≥ 75	1.571 (0.657–3.758)	0.23	0.32
Male	1.362 (1.292–1.437)	< 0.01	< 0.01
Race			
White	—	—	—
Black	0.810 (0.706–0.928)	< 0.01	< 0.01
Other race	0.969 (0.792–1.185)	0.95	0.95
Acute care indicators			
Critical care time	1.943 (1.679–2.247)	< 0.01	< 0.01
ABG/VBG	1.401 (1.199–1.638)	< 0.01	< 0.01
Intubation, endotracheal, emergency procedure	0.947 (0.740–1.211)	0.66	0.71
Ventilation	1.195 (0.892–1.601)	0.23	0.32
Pulmonary comorbidities			
History of pneumothorax	—	—	—
Other COPD	0.786 (0.653–0.947)	0.01	0.02
Emphysema	1.555 (1.198–2.018)	< 0.01	< 0.01
Asthma	0.861 (0.647–1.146)	0.31	0.41
Other interstitial pulmonary diseases	2.130 (1.630–2.781)	< 0.01	< 0.01
Acute diagnoses			
ARDS	1.305 (0.908–1.876)	0.15	0.23
Acute respiratory failure	1.227 (1.049–1.436)	0.01	0.02
Sepsis, unspecified organism	1.062 (0.895–1.262)	0.49	0.58
Other comorbidities			
Nicotine dependence	1.273 (1.084–1.492)	< 0.01	0.02
Hypertensive diseases	0.965 (0.853–1.092)	0.57	0.64
Chronic ischemic heart disease	1.266 (1.091–1.469)	< 0.01	< 0.01
Heart failure	1.051 (0.899–1.230)	0.53	0.62
Overweight or obesity	0.833 (0.663–1.046)	0.12	0.19

*Note:* The HRs of the covariates included in the Cox regression, excluding antibiotics, are shown here. The “*p* value” column shows unadjusted *p* values from Cox regression, and the “*Q* value” column shows *p* values for individual antibiotics adjusted for false discovery rate by the Benjamini–Hochberg procedure. Any values of *Q* ≤ 0.05 represent statistically significant values.

Sensitivity analyses using varying follow‐up durations demonstrated robustness of the primary findings. Analyses using 30‐ and 90‐day follow‐up windows produced effect estimates similar to those observed in the primary 60‐day analysis. Fluoroquinolones as a class were not associated with pneumothorax risk at either time point, whereas ciprofloxacin remained associated with increased risk. Overall, the direction, magnitude, and statistical significance of the associations were unchanged (Tables S1–S4). In a 1:1 propensity score‐matched analysis, 85 336 patients were retained in each cohort (Table S5). Baseline characteristics were well balanced after matching. Pneumothorax occurred in 305 (0.36%) fluoroquinolone‐treated patients and 521 (0.61%) nonfluoroquinolone‐treated patients, corresponding to a risk ratio of 0.59 (95% CI 0.51–0.67; *p* < 0.01; Table S6). Mortality was the same between groups (Table S7).

## 4 Discussion

In this large, multicenter US cohort of adults hospitalized with pneumonia, the use of fluoroquinolones as a class was not associated with an increased 60‐day risk of pneumothorax compared with nonfluoroquinolone regimens after adjustment for demographics, comorbidities, and illness severity markers. Similar results were seen when using alternative follow‐up windows of 30 and 90 days.

Previous literature has associated fluoroquinolone exposure with connective tissue dysfunction, including tendonitis and tendon rupture, aortic aneurysm and dissection, and retinal detachment [17]. The pleura is immunologically and metabolically responsive to its environment, particularly in the case of systemic or pulmonary inflammatory processes [30]. Clinically, increased odds of having taken fluoroquinolones prior to developing pneumothorax were seen in a previous case‐time‐control study in a smaller French cohort [31]. Due to the nature of their experimental design, however, they concluded that this effect was likely explained by confounding by indication or illness severity, rather than an actual increase in risk imparted by fluoroquinolones. To date, however, no other studies have examined this relationship. Our present study not only examines fluoroquinolones but also accounts for the impact of severity‐of‐illness markers, which may explain our differing results.

When examining individual fluoroquinolones, ciprofloxacin showed a higher hazard of pneumothorax, whereas levofloxacin and moxifloxacin did not. There is ex vivo mechanistic evidence that fluoroquinolones increase MMP activity [14]. However, the lack of concordant change in the clinical risk of pneumothorax across all fluoroquinolones suggests a more complex interplay of factors that may not yet be fully elucidated. On a biochemical level, there is differential MMP expression from each fluoroquinolone in question, which may contribute to this finding [32]. It is also possible that the apparent increased risk of spontaneous pneumothorax associated with ciprofloxacin may be secondary to characteristics of the underlying infection or even the host, rather than the drug itself [31, 33, 34, 35]. As a distinctly antipseudomonal quinolone, confounding by indication may also have contributed to the observed result with ciprofloxacin. However, we would expect a similar association with levofloxacin, which was not seen in our study.

Interestingly, pooled nonfluoroquinolone antibiotics were associated with a modestly reduced hazard of pneumothorax (HR 0.79, 95% CI 0.69–0.91, *Q* < 0.01). Although statistically significant, the CI approached unity, and this effect was small in absolute terms (0.35% vs. 0.54% incidence), suggesting limited clinical relevance. Moreover, the pooled association may be reflective of heterogeneity within the comparator group: Only azithromycin demonstrated a significant reduction in risk, whereas amoxicillin, ceftriaxone, and doxycycline did not. However, although fluoroquinolones as a class were not associated with increased risk of pneumothorax, comparison between class‐specific HRs demonstrated a statistically significant difference between antibiotic classes (ratio of HRs 1.63, 95% CI 1.16–2.29). This finding does not provide evidence that fluoroquinolones increased pneumothorax risk but rather suggests that nonfluoroquinolone antibiotics exhibited an overall protective association whereas fluoroquinolones did not. Propensity score matching yielded a lower observed incidence of pneumothorax among fluoroquinolone recipients. Because this finding was not the primary analysis and lacks biologic plausibility, it should be interpreted with caution. Our results nevertheless support the broader conclusion that fluoroquinolones do not appear to confer an increased risk of pneumothorax in hospitalized patients treated for community acquired pneumonia. Thus, the apparent protective association of nonfluoroquinolones is unlikely to be a uniform drug class effect.

To our knowledge, there are no studies demonstrating an association between azithromycin use and reduced risk for pneumothorax. However, its use in other diseases increases plausibility for this apparent effect. Azithromycin has well‐documented anti‐inflammatory properties in clinical studies, including those examining infectious and other inflammatory pulmonary processes [14], which may have contributed to azithromycin reducing pleural insult in our cohort. Alternatively, the apparent protective effect of azithromycin may reflect differences in prescribing patterns, as azithromycin is often used in the context of lower disease severity or a lower comorbidity burden [13].

As a secondary analysis, mortality was higher among patients receiving fluoroquinolones in the unmatched cohorts. However, this difference was no longer observed after 1:1 PSM, suggesting that baseline differences likely contributed to the crude mortality imbalance. Although residual confounding cannot be excluded, this finding suggests that the observed mortality difference was largely due to differences in baseline patient characteristics and aligns with existing literature examining mortality of patients with community acquired pneumonia treated with fluoroquinolones versus other antibiotic classes [36, 37]. Other factors associated with an increased risk of pneumothorax in our analyses were consistent with the existing literature, including younger age, male sex, emphysema, and interstitial lung disease [38].

This study has several strengths compared with prior work assessing fluoroquinolones' effect on the risk of pneumothorax. The large US‐based multi‐institutional cohort provided statistical power and generalizability. Restricting the population to adults with pneumonia and hospitalization and defining cohorts by initial antibiotic regimen for community acquired pneumonia reduced confounding by indication. Reducing antibiotic prescription on the same day as the pneumonia diagnosis increased temporal alignment between the exposure and outcome. Compared with the prior case‐time‐control study [30], we compared fluoroquinolones class‐wise and individually to other common pneumonia treatments, adjusted for a broader set of covariates, and used a retrospective cohort design with an adjusted Cox model for a time‐to‐event analysis. Notably, the direction of the findings was consistent across the primary multivariable Cox regression, propensity score‐matched analysis, and sensitivity analyses using alternative follow‐up windows. Collectively, these analyses failed to demonstrate an increased risk of pneumothorax associated with the fluoroquinolone drug class as a whole.

Limitations include those shared with all TriNetX data: reliance on ICD codes, which may be confounded by incomplete capture, miscoding, or misclassification; lack of radiological studies; and incomplete medication details such as dose, timing, or postdischarge adherence. Due to limitations imposed by the dataset, we were unable to assess for dose–response relationships. Furthermore, unmeasured factors that influenced the antibiotic choice, such as microbiological results, allergies, and local resistance patterns, were not available. Additionally, our cohort had a relatively low incidence of pneumothorax relative to the total sample size, which may have impacted study power. Compared with the prior case‐time‐control using a within‐person design, our approach with a between‐person design is more susceptible to residual confounding. Although baseline characteristics were adjusted for, residual confounding from unmeasured variables may persist. We did find that mortality exceeded 10% in both groups in the follow up windows, but the magnitude of the mortality difference between cohorts was modest. Implementation of the Fine‐Gray subdistribution hazard model was not supported within the TriNetX platform. Therefore, the competing risk of mortality cannot be excluded.

## 5 Conclusions

In this large US cohort of patients hospitalized with community acquired pneumonia, we found that fluoroquinolones as a class were not associated with a higher incidence of pneumothorax. Future work may involve longitudinal, prospective follow‐up of patients hospitalized with pneumonia to better assess other potential confounders. Access to information on radiographic reports, coincident procedures, and pneumonia severity scores could also inform future prescribing practices. Ultimately, mechanistic research in both clinical and experimental settings may help elucidate whether the mechanism by which fluoroquinolones affect connective tissue, such as the pulmonary pleura, varies by acute disease state.

## Author Contributions

S.Y. and S.S. had full access to all the data and take responsibility for the integrity of the data and the accuracy of the data analysis. S.Y., S.S., J.C., and E.J.Y. contributed substantially to the conceptualization, study design, methodology, data validation and interpretation, visualization, and the writing of the manuscript.

## Funding

The authors have nothing to report.

## Conflicts of Interest

The authors declare no conflicts of interest.

## Supporting information


**Table S1:** Hazard ratios for 30‐day pneumothorax risk sensitivity analysis. Adjusted hazard ratios (HRs) and 95% confidence intervals (CIs) of the baseline clinical and demographic covariates derived from multivariable Cox proportional hazards regression modeling are presented, excluding specific antibiotic variables. Unadjusted *p* values reflect independent Wald tests within the regression model. A two‐tailed *Q <* 0.05 denotes statistical significance. *Abbreviations:* HR, hazard ratio; CI, confidence interval; COPD, chronic obstructive pulmonary disease; ARDS, acute respiratory distress syndrome; ABG, arterial blood gas; VBG, venous blood gas.

## Data Availability

The data that support the findings of this study are available from the corresponding author upon reasonable request.

## References

[1] B. H. Hayes , D. L. Haberling , J. L. Kennedy , J. K. Varma , A. M. Fry , and N. M. Vora , “Burden of Pneumonia‐Associated Hospitalizations,” Chest 153 (2018): 427–437, 10.1016/j.chest.2017.09.041. 29017956 PMC6556777

[2] E. Ekanem , S. Podder , N. Donthi , et al., “Spontaneous Pneumothorax: An Emerging Complication of COVID‐19 Pneumonia,” Heart & Lung: The Journal of Critical Care 50 (2021): 437–440, 10.1016/j.hrtlng.2021.01.020. 33631467 PMC7846243

[3] S. Chandna , K. Raj , A. Agrawal , et al., “Prevalence and Patient Risk Factors for Pneumothorax in COVID‐19 and in Influenza Pneumonia: A Nationwide Comparative Analysis,” Journal of Thoracic Disease 16 (2024): 3593–3605, 10.21037/jtd-23-1454. 38983184 PMC11228741

[4] J. Ludwig and G. D. Kienzle , “Pneumothorax in a Large Autopsy Population. A Study of 77 Cases,” American Journal of Clinical Pathology 70 (1978): 24–26, 10.1093/ajcp/70.1.24. 696669

[5] F. J. de Latorre , A. Tomasa , J. Klamburg , C. Leon , M. Soler , and J. Rius , “Incidence of Pneumothorax and Pneumomediastinum in Patients With Aspiration Pneumonia Requiring Ventilatory Support,” Chest 72 (1977): 141–144, 10.1378/chest.72.2.141. 884974

[6] S. Nishizawa , K. Tobino , Y. Murakami , et al., “Mortality and Prognostic Factors for Spontaneous Pneumothorax in Older Adults,” PLoS ONE 18 (2023): e0291233, 10.1371/journal.pone.0291233. 37682952 PMC10490947

[7] J. Akram , Z. Yousaf , Y. Alabbas , M. I. A. Almoyaaf , A. S. S. Ibrahim , and N. Kharma , “Epidemiological and Outcome Analysis of COVID‐19‐Associated Pneumothorax: Multicentre Retrospective Critical Care Experience From Qatar,” BMJ Open 12 (2022): e053398, 10.1136/bmjopen-2021-053398. PMC8889440 35190427

[8] A. Nasrullah , M. A. Quazi , S. Virk , et al., “Impact of Pneumothorax on Mortality, Morbidity, and Hospital Resource Utilization in COVID‐19 Patients: A Propensity Matched Analysis of Nationwide Inpatient Sample Database,” BMC Pulmonary Medicine 24 (2024): 371, 10.1186/s12890-024-03161-z. 39085906 PMC11293109

[9] N.‐C. Huan , C. Sidhu , and R. Thomas , “Pneumothorax: Classification and Etiology,” Clinics in Chest Medicine 42 (2021): 711–727, 10.1016/j.ccm.2021.08.007. 34774177

[10] C. Mauch , T. Krieg , and E. A. Bauer , “Role of the Extracellular Matrix in the Degradation of Connective Tissue,” Archives of Dermatological Research 287 (1994): 107–114, 10.1007/BF00370728. 7726628

[11] C.‐K. Chen , P.‐R. Chen , H.‐C. Huang , Y.‐S. Lin , and H.‐Y. Fang , “Overexpression of Matrix Metalloproteinases in Lung Tissue of Patients With Primary Spontaneous Pneumothorax,” Respiration: International Review of Thoracic Diseases 88 (2014): 418–425, 10.1159/000366065. 25300296

[12] C.‐Y. Chiu , T.‐P. Chen , J.‐R. Chen , et al., “Overexpression of Matrix Metalloproteinase‐9 in Adolescents With Primary Spontaneous Pneumothorax for Surgical Intervention,” Journal of Thoracic and Cardiovascular Surgery 156 (2018): 2328–2336.e2, 10.1016/j.jtcvs.2018.05.083. 30033106

[13] M. Hameed , W. Jamal , M. Yousaf , et al., “Pneumothorax in Covid‐19 Pneumonia: A Case Series,” Respiratory Medicine Case Reports 31 (2020): 101265, 10.1016/j.rmcr.2020.101265. 33101895 PMC7576439

[14] D. G. Guzzardi , G. Teng , S. Kang , et al., “Induction of Human Aortic Myofibroblast‐Mediated Extracellular Matrix Dysregulation: A Potential Mechanism of Fluoroquinolone‐Associated Aortopathy,” Journal of Thoracic and Cardiovascular Surgery 157 (2019): 109–119.e2, 10.1016/j.jtcvs.2018.08.079. 30528439

[15] J. Liu , W. Xiong , L. Baca‐Regen , H. Nagase , and B. T. Baxter , “Mechanism of Inhibition of Matrix Metalloproteinase‐2 Expression by Doxycycline in Human Aortic Smooth Muscle Cells,” Journal of Vascular Surgery 38 (2003): 1376–1383, 10.1016/s0741-5214(03)01022-x. 14681644

[16] S.‐C. Hsu , S.‐S. Chang , M. G. Lee , et al., “Risk of Gastrointestinal Perforation in Patients Taking Oral Fluoroquinolone Therapy: An Analysis of Nationally Representative Cohort,” PLoS ONE 12 (2017): e0183813, 10.1371/journal.pone.0183813. 28873440 PMC5584983

[17] X. Yu , D. Jiang , J. Wang , et al., “Fluoroquinolone Use and the Risk of Collagen‐Associated Adverse Events: A Systematic Review and Meta‐Analysis,” Drug Safety 42 (2019): 1025–1033, 10.1007/s40264-019-00828-z. 31077091

[18] B. Pasternak , M. Inghammar , and H. Svanström , “Fluoroquinolone Use and Risk of Aortic Aneurysm and Dissection: Nationwide Cohort Study,” BMJ 360 (2018): k678, 10.1136/bmj.k678. 29519881 PMC5842359

[19] S. Baik , J. Lau , V. Huser , et al., “Association Between Tendon Ruptures and Use of Fluoroquinolone, and Other Oral Antibiotics: A 10‐Year Retrospective Study of 1 Million US Senior Medicare Beneficiaries,” BMJ Open 10 (2020): e034844, 10.1136/bmjopen-2019-034844. PMC7754651 33371012

[20] T.‐Y. Chiang , S.‐M. Tsao , C.‐B. Yeh , and S.‐F. Yang , “Matrix Metalloproteinases in Pneumonia,” Clinica Chimica Acta: International Journal of Clinical Chemistry 433 (2014): 272–277, 10.1016/j.cca.2014.03.031. 24721641

[21] TriNetX , “Datasets—Build and License From Global RWD [Dataset],” (2026) TriNetX.

[22] J. P. Metlay , G. W. Waterer , A. C. Long , et al., “Diagnosis and Treatment of Adults With Community‐Acquired Pneumonia,” American Journal of Respiratory and Critical Care Medicine 200, no. 7 (2019): e45–e67, 10.1164/rccm.201908-1581ST. 31573350 PMC6812437

[23] Python Software Foundation , Python 3.13 Documentation (Python Software Foundation, accessed September 25, 2025) https://docs.python.org/3/.

[24] The Pandas Development Team , pandas‐dev/pandas: Pandas2025 (Pandas Development Team, 2025), 10.5281/zenodo.16918803.

[25] P. Virtanen , R. Gommers , T. E. Oliphant , et al., “SciPy 1.0: Fundamental Algorithms for Scientific Computing in Python,” Nature Methods 17 (2020): 261–272, 10.1038/s41592-019-0686-2. 32015543 PMC7056644

[26] J. D. Hunter , “Matplotlib: A 2D Graphics Environment,” Computing in Science & Engineering 9 (2007): 90–95, 10.1109/MCSE.2007.55.

[27] S. Lsy , J. Q. Aguilera , and A. Shapiro , LSYS/Forestplot: v0.4.12024 (Zenodo), 10.5281/zenodo.13118398.

[28] A. A. Veroniki and J. E. McKenzie , “A Brief Note on the Common (Fixed)‐Effect Meta‐Analysis Model,” Journal of Clinical Epidemiology 169 (2024): 111281, 10.1016/j.jclinepi.2024.111281. 38364875

[29] D. Y. Lin , L. Dai , G. Cheng , and M. O. Sailer , “On Confidence Intervals for the Hazard Ratio in Randomized Clinical Trials,” Biometrics 72 (2016): 1098–1102, 10.1111/biom.12528. 27123760 PMC5085885

[30] V. B. Antony , “Immunological Mechanisms in Pleural Disease,” European Respiratory Journal 21 (2003): 539–544, 10.1183/09031936.03.00403902. 12662014

[31] A. Bénard‐Laribière , E. Pambrun , S. Kouzan , J.‐L. Faillie , J. Bezin , and A. Pariente , “Association of Fluoroquinolones With the Risk of Spontaneous Pneumothorax: Nationwide Case‐Time‐Control Study,” Thorax 80 (2025): 159–166, 10.1136/thorax-2024-221779. 39393909 PMC11877017

[32] R. Hallifax , “Pneumothorax and Antibiotic Use: A Clue to Aetiology of Primary Spontaneous Pneumothorax?,” Thorax 80 (2025): 129–130, 10.1136/thorax-2024-222542. 39939169

[33] A. P. MacGowan , C. A. Rogers , H. A. Holt , and K. E. Bowker , “Activities of Moxifloxacin Against, and Emergence of Resistance in, *Streptococcus pneumoniae* and *Pseudomonas aeruginosa* in an In Vitro Pharmacokinetic Model,” Antimicrobial Agents and Chemotherapy 47 (2003): 1088–1095, 10.1128/aac.47.3.1088-1095.2003. 12604546 PMC149285

[34] K. S. Kaye , Z. A. Kanafani , A. E. Dodds , J. J. Engemann , S. G. Weber , and Y. Carmeli , “Differential Effects of Levofloxacin and Ciprofloxacin on the Risk for Isolation of Quinolone‐Resistant *Pseudomonas aeruginosa* ,” Antimicrobial Agents and Chemotherapy 50 (2006): 2192–2196, 10.1128/aac.00060-06. 16723582 PMC1479159

[35] W. M. Scheld , “Maintaining Fluoroquinolone Class Efficacy: Review of Influencing Factors,” Emerging Infectious Diseases 9, no. 1 (2003): 1–9, 10.3201/eid0901.020277. 12533274 PMC2873754

[36] S. Liu , X. Tong , Y. Ma , et al., “Respiratory Fluoroquinolones Monotherapy vs. β‐Lactams With or Without Macrolides for Hospitalized Community‐Acquired Pneumonia Patients: A Meta‐Analysis,” Frontiers in Pharmacology 10 (2019): 489, 10.3389/fphar.2019.00489. 31139081 PMC6517694

[37] S. H. Choi , A. Cesar , T. A. C. Snow , et al., “Respiratory Fluoroquinolone Monotherapy vs. β‐Lactam Plus Macrolide Combination Therapy for Hospitalized Adults With Community‐Acquired Pneumonia: A Systematic Review and Meta‐Analysis of Randomized Controlled Trials,” International Journal of Antimicrobial Agents 62, no. 1 (2023): 106905. 37385561 10.1016/j.ijantimicag.2023.106905

[38] R. J. Hallifax , R. Goldacre , M. J. Landray , N. M. Rahman , and M. J. Goldacre , “Trends in the Incidence and Recurrence of Inpatient‐Treated Spontaneous Pneumothorax, 1968‐2016,” JAMA 320 (2018): 1471–1480, 10.1001/jama.2018.14299. 30304427 PMC6233798

