# Supplementary material for: Fluoroquinolone Use and Risk of Pneumothorax in Adults Hospitalized With Community Acquired Pneumonia: A Retrospective Cohort Study
Source: Clin Respir J. 2026 Jul 1;20(7):e70212. doi: 10.1111/crj.70212 (PMC13323163; doi:10.1111/crj.70212)
Supplement: Supplementary file 1 — Table S1: Hazard ratios for 30‐day pneumothorax risk sensitivity analysis. Adjusted hazard ratios (HRs) and 95% confidence intervals (CIs) of the baseline clinical and demographic covariates derived from multivariable Cox proportional hazards regression modeling are presented, excluding specific antibiotic variables. Unadjusted p values reflect independent Wald tests within the regression model. A two‐tailed Q < 0.05 denotes statistical significance. Abbreviations: HR, hazard ratio; CI, confidence interval; COPD, chronic obstructive pulmonary disease; ARDS, acute respiratory distress syndrome; ABG, arterial blood gas; VBG, venous blood gas. [file CRJ-20-e70212-s001.docx]

|  | Hazard Ratio (95% CI) | P value | Q value |
| --- | --- | --- | --- |
| Demographics |  |  |  |
| Age |  |  |  |
| <65 Years | 2.867  (1.074, 7.649) | 0.04 | 0.14 |
| 65–75 Years | 2.222  (0.937, 6.685) | 0.11 | 0.23 |
| >75 | 1.387  (0.519, 3.707) | 0.51 | 0.70 |
| Male | 1.251  (1.170, 1.339) | <0.01 | <0.01 |
| Race |  |  |  |
| White | - | - | - |
| Black | 1.005  (0.838-1.206) | 0.95 | 0.97 |
| Other race | 1.100  (1.228, 1.691) | 0.47 | 0.64 |
| Acute Care Indicators |  |  |  |
| Critical Care Time | 1.365  (1.109, 1.681) | <0.01 | 0.02 |
| ABG/VBG | 1.693  (1.420-2.020) | <0.01 | <0.01 |
| Intubation, endotracheal, emergency procedure | 1.261  (0.963-1.651) | 0.09 | 0.21 |
| Ventilation | 1.422  (1.041-1.943) | 0.03 | 0.11 |
| Pulmonary Comorbidities |  |  |  |
| History of Pneumothorax |  |  |  |
| Other COPD | 0.753  (0.600-0.945) | 0.01 | 0.04 |
| Emphysema | 1.457  (1.054-2.013) | 0.02 | 0.09 |
| Asthma | 0.934  (0.672-1.300) | 0.69 | 0.83 |
| Other interstitial pulmonary diseases | 1.951  (1.402-2.713) | <0.01 | <0.01 |
| Acute Diagnoses |  |  |  |
| ARDS | 1.102  (0.699-1.738) | 0.67 | 0.83 |
| Acute respiratory failure | 1.563  (1.307-1.869) | 0.09 | 0.21 |
| Sepsis, unspecified organism | 1.117  (0.913-1.367) | 0.88 | 0.93 |
| Other Comorbidities |  |  |  |
| Nicotine dependence | 1.002  (0.817-1.228) | 0.99 | 0.99 |
| Hypertensive diseases | 0.932  (0.801-1.086) | 0.37 | 0.57 |
| Chronic ischemic heart disease | 1.126  (0.936-1.365) | 0.20 | 0.33 |
| Heart failure | 1.131  (0.937-1.365) | 0.20 | 0.35 |
| Overweight or obesity | 0.968  (0.743-1.262) | 0.81 | 0.88 |

**Table S1:**

**Supplemental Table 1. Hazard ratios for 30-day pneumothorax risk sensitivity analysis.** Adjusted hazard ratios (HRs) and 95% confidence intervals (CIs) of the baseline clinical and demographic covariates derived from multivariable Cox proportional hazards regression modeling are presented, excluding specific antibiotic variables. Unadjusted *P*-values reflect independent Wald tests within the regression model. A two-tailed *Q <* 0.05 denotes statistical significance.

*Abbreviations:* HR, hazard ratio; CI, confidence interval; COPD, chronic obstructive pulmonary disease; ARDS, acute respiratory distress syndrome; ABG, arterial blood gas; VBG, venous blood gas.

|  | Hazard Ratio (95% CI) | P value | Q value |
| --- | --- | --- | --- |
| Demographics |  |  |  |
| Age |  |  |  |
| <65 Years | 3.094  (1.285-7.447) | 0.01 | 0.02 |
| 65–75 Years | 2.398  (0.988-5.726) | 0.04 | 0.08 |
| >75 | 1.401  (0.582-3.374) | 0.32 | 0.51 |
| Male | 1.377  (1.307-1.450) | <0.01 | <0.01 |
| Race |  |  |  |
| White | - | - | - |
| Black | 0.777  (0.679-0.889) | <0.01 | <0.01 |
| Other Race | 0.969  (0.792, 1.185) | 0.76 | 0.78 |
| Acute Care Indicators |  |  |  |
| Critical Care Time | 1.530  (1.298, 1.802) | <0.01 | <0.01 |
| ABG/VBG | 1.631  (1.402-1.897) | <0.01 | <0.01 |
| Intubation, endotracheal, emergency procedure | 1.290  (1.009-1.650) | 0.04 | 0.08 |
| Ventilation | 1.227  (0.919-1.638) | 0.17 | 0.31 |
| Pulmonary Comorbidities |  |  |  |
| History of Pneumothorax |  |  |  |
| Other COPD | 0.763  (0.633-0.919) | <0.01 | <0.01 |
| Emphysema | 1.461  (1.113-1.916) | <0.01 | <0.01 |
| Asthma | 0.597  (0.439-0.812) | <0.01 | <0.01 |
| Other interstitial pulmonary diseases | 1.926  (1.465-2.532) | <0.01 | <0.01 |
| Acute Diagnoses |  |  |  |
| ARDS | 1.614  (1.139-2.287) | 0.01 | 0.02 |
| Acute respiratory failure | 1.322  (1.129-1.548) | <0.01 | <0.01 |
| Sepsis, unspecified organism | 1.049  (0.880-1.249) | 0.59 | 0.87 |
| Other Comorbidities |  |  |  |
| Nicotine dependence | 0.933  (0.784-1.109) | 0.43 | 0.66 |
| Hypertensive diseases | 0.965  (0.855-1.090) | 0.57 | 0.87 |
| Chronic ischemic heart disease | 1.326  (1.145-1.535) | <0.01 | <0.01 |
| Heart failure | 1.074  (0.920-1.255) | 0.37 | 0.59 |
| Overweight or obesity | 0.815  (0.648-1.026) | 0.08 | 0.15 |

**Table S2:**

**Supplemental Table 2. Hazard ratios for 90-day pneumothorax risk sensitivity analysis.** Adjusted hazard ratios (HRs) and 95% confidence intervals (CIs) for baseline demographic characteristics, acute clinical indices, and underlying comorbidities are displayed over an extended 90-day post-index observational window. Estimates were calculated via multivariable Cox proportional hazards regression excluding structural antibiotic covariates. The displayed *P*-values indicate unadjusted regression significance levels. A two-tailed *Q <* 0.05 denotes statistical significance.

*Abbreviations:* HR, hazard ratio; CI, confidence interval; COPD, chronic obstructive pulmonary disease; ARDS, acute respiratory distress syndrome; ABG, arterial blood gas; VBG, venous blood gas.

|  | Hazard Ratio (95% CI) | P value | Q value |
| --- | --- | --- | --- |
| No FQ exposure |  |  |  |
| Amoxicillin | 0.733  (0.466-1.154) | 0.18 | 0.30 |
| Azithromycin | 0.706  (0.517-0.965) | 0.03 | 0.06 |
| Ceftriaxone | 0.881  (0.696-1.116) | 0.29 | 0.44 |
| Doxycycline | 0.955  (0.678-1.345) | 0.79 | 0.86 |
| FQ Exposure |  |  |  |
| Moxifloxacin | 1.529  (0.567-4.126) | 0.40 | 0.55 |
| Ciprofloxacin | 2.353  (1.362-4.064) | <0.01 | <0.01 |
| Levofloxacin | 0.751  (0.567-1.256) | 0.28 | 0.42 |
| Ofloxacin | - | - | - |

**Table S3:**

**Supplemental Table 3: 30-day pneumothorax hazard risks stratified by pooled cohort and individual antibiotic exposure.** Adjusted hazard ratios (HRs) and corresponding 95% confidence intervals (CIs) describe the time-to-event liability of incident pneumothorax within a localized 30-day window following initial pneumonia diagnosis and acute hospitalization. A *Q*-value 0.05 is interpreted as statistically significant. *Abbreviations:* HR, hazard ratio; CI, confidence interval; FQ, fluoroquinolone.

|  | Hazard Ratio (95% CI) | P value | Q value |
| --- | --- | --- | --- |
| Non-FQ exposure |  |  |  |
| Amoxicillin | 0.855  (0.615-1.190) | 0.35 | 0.47 |
| Azithromycin | 0.667  (0.520, 0.965) | <0.01 | <0.01 |
| Ceftriaxone | 0.943  (0.774-1.148) | 0.56 | 0.68 |
| Doxycycline | 0.854  (0.642-1.138) | 0.28 | 0.40 |
| FQ Exposure |  |  |  |
| Moxifloxacin | 0.822  (0.339-1.991) | 0.66 | 0.76 |
| Ciprofloxacin | 2.213  (1.374-3.564) | <0.01 | <0.01 |
| Levofloxacin | 1.005  (0.681-1.482) | 0.98 | 0.98 |
| Ofloxacin | - | - | - |

**Table S4:**

**Supplemental Table 4: 90-day pneumothorax hazard risks stratified by pooled cohort and individual antibiotic exposure.** Adjusted hazard ratios (HRs) and 95% confidence intervals (CIs) characterize the long-term relative hazard of incident pneumothorax over a 90-day longitudinal follow-up period. A *Q*-value 0.05 is interpreted as statistically significant. *Abbreviations:* HR, hazard ratio; CI, confidence interval; FQ, fluoroquinolone.

| **Characteristic** | **FQ After Matching (n=85,336)** | **Non-FQ After Matching (n=85,336)** | **SMD After** |
| --- | --- | --- | --- |
| Age, mean ± SD | 67.3 ± 16.7 | 67.8 ± 16.7 | 0.027 |
| White race | 60,930(71.4%) | 61442  (72.0%) | 0.015 |
| Male sex | 42,071  (49.3%) | 42071  (49.3%) | 0.001 |
| Hypertension | 44,545  (52.2%) | 45,228  (53.0%) | 0.016 |
| Ischemic heart disease | 21,419  (25.1%) | 21,675  (25.4%) | 0.006 |
| COPD | 17,067  (20.0%) | 17,236  (20.2%) | 0.004 |
| Heart failure | 15,958  (18.7%) | 15,781  (18.5%) | 0.005 |
| Respiratory failure | 14,251  (16.7%) | 13,563  (15.9%) | 0.023 |
| Nicotine dependence | 14,083  (16.5%) | 14,251  (16.7%) | 0.006 |
| Obesity | 11,352  (13.3%) | 11,528  (13.5%) | 0.006 |
| Asthma | 6,998  (8.2%) | 6,917  (8.1%) | 0.004 |
| Emphysema | 4,523  (5.3%) | 4,351  (5.1%) | 0.009 |
| ILD | 2,816  (3.3%) | 2554  (3.0%) | 0.02 |
| ARDS | 939  (1.1%) | 857  (1.0%) | 0.014 |
| Prior pneumothorax | 427  (0.5%) | 341  (0.4%) | 0.008 |
| Critical care services | 10,411  (12.2%) | 10,070  (11.8%) | 0.012 |
| Emergency intubation | 1,963  (2.3%) | 1,621  (1.9%) | 0.029 |

**Table S5:**

**Supplemental Table 5: Propensity score matching and pneumothorax outcomes**. Baseline characteristics of patients receiving fluoroquinolone (FQ) and non-fluoroquinolone (non-FQ) antibiotic regimens for pneumonia before and after 1:1 propensity score matching are shown. Matching was performed using demographic characteristics, comorbidities, markers of illness severity, and prior pneumothorax history. After matching, 85,336 patients remained in each cohort with insignificant standardized mean differences.

|  | **FQ Exposure** | **Non-FQ Exposure** | **Hazard Ratio (HR)** | **P-value** |
| --- | --- | --- | --- | --- |
| **Before Matching (Crude)** | N = 86,814 | N = 1,062,657 |  |  |
| Event Count (n) | 302 | 5,678 | — | — |
| Absolute Risk (%) | 0.35% | 0.53% | — | — |
| Cox Hazard Ratio | — | — | HR: 0.607  (0.541, 0.682) | <0.01 |
| **After 1:1 PSM (Balanced)** | N = 85,336 | N = 85,336 |  |  |
| Event Count (n) | 305 | 521 | — | — |
| Absolute Risk (%) | 0.36% | 0.61% | — | — |
| Risk Ratio | — | — | HR: 0.585  (0.508, 0.674) | < 0.01 |

**Table S6:**

**Supplemental Table 6: Raw pneumothorax rates within follow-up windows across antibiotic exposure cohorts.** Observed all-cause mortality counts and crude percentages are presented for the fluoroquinolone (FQ) and non-fluoroquinolone (non-FQ) cohorts assessed within 60 days of initiation of the antibiotic regimens. Before 1:1 PSM, and after 1:1 PSM, the fluoroquinolone group experienced a lower rate of pneumothorax incidence.

*Abbreviations*. FQ = fluoroquinolone

|  | **FQ Exposure** | **Non-FQ Exposure** | **Hazard Ratio (HR)** | **P-value** |
| --- | --- | --- | --- | --- |
| **Before Matching (Crude)** | N = 86,591 | N = 1,053,515 |  |  |
| Event Count (n) | 12,583 | 12,547 | — | — |
| Absolute Risk (%) | 14.90% | 14.10% | — | — |
| Cox Hazard Ratio | — | — | 1.084  (1.064, 1.104) | <0.01 |
| **After 1:1 PSM (Balanced)** | N = 85,336 | N = 85,336 |  |  |
| Event Count (n) | 13,408 | 13,371 | — | — |
| Absolute Risk (%) | 15.71% | 15.67% | — | — |
| Cox Hazard Ratio | — | — | 1.004  (0.980, 1.028) | 0.32 |

**Table S7:**

**Supplemental Table 7: Raw mortality rates within follow-up windows across antibiotic exposure cohorts.** Observed all-cause mortality counts and crude percentages are presented for the fluoroquinolone (FQ) and non-fluoroquinolone (non-FQ) cohorts assessed within 60 days of initiation of the antibiotic regimens. Before 1:1 PSM, the fluoroquinolone group faced slightly higher mortality. However, after 1:1 PSM, the groups did not encounter significantly different mortality. Note: Due to constraints of the TriNetX analytics platform, implementation of a Fine-Gray proportional subdistribution hazards model or equivalent competing-risk framework was not supported. Consequently, all-cause mortality could not be formally modeled as a competing event for incident pneumothorax, and residual selection or survivorship biases may persist.

*Abbreviations*. FQ = fluoroquinolone
